# Relation of Serum Hepcidin Levels and Restless Legs Syndrome in Patients Undergoing Peritoneal Dialysis

**DOI:** 10.3389/fmed.2021.685601

**Published:** 2021-12-13

**Authors:** Yanhong Guo, Yuan Sang, Tian Pu, Xiaodan Li, Yulin Wang, Lu Yu, Yan Liang, Liuwei Wang, Peipei Liu, Lin Tang

**Affiliations:** ^1^Department of Nephropathy, The First Affiliated Hospital of Zhengzhou University, Zhengzhou, China; ^2^Department of Gastroenterology, The First Affiliated Hospital of Zhengzhou University, Zhengzhou, China; ^3^Clinical Systems Biology Laboratories, The First Affiliated Hospital of Zhengzhou University, Zhengzhou, China

**Keywords:** restless leg syndrome (RLS), hepcidin, peritoneal dialysis, ROC (receiver operating characteristic curve), IRLS

## Abstract

**Introduction:** Restless legs syndrome is a common and severe complication in patients undergoing peritoneal dialysis (PD), which seriously affects the life quality and prognosis of patients undergoing PD. Unfortunately, there are still no effective prevention and treatment measures. Serum hepcidin was demonstrated to be related to primary restless legs syndrome (RLS), whereas there are no studies on the relationship between serum hepcidin and RLS in patients undergoing PD. We aimed to evaluate the role and function of serum hepcidin in patients undergoing PD with RLS.

**Methods:** A total of 51 patients undergoing PD with RLS and 102 age-and gender-matched patients undergoing PD without RLS were included. We collected the clinical data including serum hepcidin of those patients undergoing PD. We scored the severity of RLS according to the International restless leg Syndrome Research Group rating scale (IRLS). We compared the clinical characteristics of the two groups and evaluated the determinant factors of RLS by Logistic regression analysis. In addition, we evaluated the diagnostic value of serum hepcidin in patients undergoing PD with RLS by receiver operating characteristic (ROC) curve. We also analyzed the influencing factors of IRLS by multivariate linear regression analysis.

**Results:** The duration of PD, serum hepcidin, and calcium were found to be significantly higher in patients undergoing PD with RLS than those patients undergoing PD without RLS (*P* < 0.001, *P* < 0.001, and *P* = 0.002, respectively). The level of hemoglobin, albumin, and RKF were significantly lower in patients undergoing PD with RLS (*P* = 0.002, *P* = 0.042, and *P* < 0.001, respectively). The duration of PD [odds ratio (OR) 1.038, 95% CI: 1.017, 1.060, *P* < 0.001], hemoglobulin level (OR 0.969, 95% CI: 0.944, 0.995, *P* = 0.019), calcium level (OR 9.224, 95% CI: 1.261, 67.450, *P* = 0.029), albumin level (OR 0.835, 95% CI: 0.757, 0.921, *P* < 0.001), hepcidin level (OR 1.023, 95% CI: 1.009, 1.038, *P* = 0.001), and RKF (OR 0.65, 95% CI: 0.495, 0.856, *P* = 0.002) are independent determinant factors of RLS in patients undergoing PD. Multivariate linear regression analysis revealed that, in addition to albumin, they were also independently associated with the severity of RLS.

**Conclusion:** A significant relation was detected between serum hepcidin level and RLS in patients undergoing PD.

## Introduction

Peritoneal dialysis (PD) is an effective treatment for patients with end-stage renal disease (ESRD). At present, more than 272,000 ESRD patients receiving peritoneal dialysis treatment worldwide, which accounts for ~11% of dialysis patients (1). Restless legs syndrome (RLS) is a neurological sensorimotor disorder and a severe complication in patients undergoing PD, with a prevalence rate of about 24.7% (2). Patients undergoing PD with RLS will feel an urge to move the legs especially during rest which will lead to having trouble falling asleep (3). RLS not only reduces the sleep quality of patients which induces daytime fatigue, poor mental health, and emotional anxiety, but also may increase the risk of hypertension, cardiovascular disease, and cerebrovascular disease (4). RLS seriously affects the quality of life and prognosis of patients undergoing PD, but still lacks effective treatment (2). It's essential to understand the pathogenesis of RLS.

At present, it is believed that dopaminergic dysfunction and the decrease of the iron level in the central nervous system play an important role in the pathogenesis of RLS (5). During the synthesis of dopamine, tyrosine hydroxylase is a key enzyme, while iron works as the coenzyme of tyrosine hydroxylase. Iron deficiency in the central nervous system will affect the synthesis and function of dopamine (6).

Serum ferritin works as the main indicator of iron storage in the body. However, due to the increase of serum ferritin secondary to inflammation and intravenous or oral iron supplements in patients undergoing PD, the level of serum ferritin does not fully reflect decreased iron stores in patients undergoing PD (7). Hepcidin has been demonstrated to be the main regulator of iron metabolism in the human body. Ferroportin 1 is an iron export protein on the cell membrane of the reticuloendothelial system, duodenal epithelial cells, and hepatocytes (8). Hepcidin can induce iron internalization and degradation, reduce iron absorption and iron storage release, and lead to an increase in intracellular iron storage and a decrease in circulating iron levels by interacting with ferroportin 1 (9). As a result, hepcidin will result in a reduction of iron utilization in circulation. There are also hepcidin and its receptor in brain tissue, and the increase of hepcidin will reduce the utilization of iron in the brain (10, 11).

The study of Dauvilliers et al. (12) have found that the serum hepcidin level was elevated in patients with idiopathic RLS compared with a control group. Serum hepcidin may be a more relevant biomarker of RLS than ferritin (13). Ahmet Tufekci et al. (14) also demonstrated that serum hepcidin was significantly associated with the presence of RLS. No studies on the relationship between serum hepcidin and RLS in patients undergoing PD were reported at present. We speculate that the increase of serum hepcidin level in patients undergoing PD leads to the disturbance of iron utilization in the central nervous system, which affects the synthesis and function of dopamine and participates in the occurrence and development of RLS. In this study, we analyzed the correlation of serum hepcidin with RLS for the first time, so as to hope to provide a new theoretical basis and therapeutic target for the treatment of RLS.

## Methods

### Study Design and Participants

A total of 51 patients undergoing PD with RLS administered in the First Affiliated Hospital of Zhengzhou University between January 2010 and December 2020 with the following criteria were included in this study: (1) All patients received PD treatment for at least 6 months and were aged ≥ 18 years at baseline; (2) All patients were diagnosed as CKD according to KDIGO guidelines (15); (3) All patients were diagnosed as RLS according to the diagnostic criteria from the International RLS Study Group (IRLSSG) (16); (4) All patients had complete clinical data. The exclusion criteria were obvious infections including peritonitis, inflammatory disease within the last 3 months, end-stage liver disease, malignancies, drug addiction, neurological diseases, and malabsorption syndromes. The patients who took drugs affecting the central nervous system or sleep or movement were excluded from the study.

Then, we randomly selected 102 patients without RLS out of a pool of 523 patients undergoing PD who were inpatients in the same period. There was no significant difference in age and gender between the two groups. The exclusion criteria of the control group were the same as those of the case group. All patients received continuous ambulatory peritoneal dialysis or automated peritoneal dialysis with glucose-based, lactate-buffered dialysis solutions (Dianeal, Baxter, China). The urea clearance index (Kt/V) was calculated using the formula recommended in the KDOQI guidelines and was used to assess the adequacy of PD (17). According to the International restless leg Research Group score scale (IRLS), the degree of RLS severity was evaluated as follows: mild (IRLS scores, 1–10), moderate (IRLS scores, 11–20), severe (IRLS scores, 21–30), and very severe (IRLS scores, 31–40) (18). The study protocol was approved by the local ethics committee, and all patients provided written informed consent.

### Clinical Data Collection

The collection of clinical data was precisely started when the expression level of hepcidin was measured. Patient demographic features such as age, gender, body weight, height, body mass index (BMI), and duration of PD were collected. The 24-h urine and dialysate samples were obtained to calculate whole body weekly urea clearance (kt/v). Furthermore, 24-h dialysate and urine volumes and the patients' weight and height were measured. Creatinine and urea nitrogen were measured in fasting blood, urine, and drained dialysate. Then, the above results were entered into the software PD adquest version 2 for Windows software (Baxter Healthcare Corporation, Chicago, IL, USA) to obtain total kt/v. Residual kidney function (RKF) was determined by residual glomerular filtration rate derived from the average of 24 h urinary urea and creatinine clearances normalized to the standard body surface area of 1.73 m (2). Serum hepcidin was measured using the Human Hepcidin ELISA kit (ZCIBIO Technology Co., Ltd, Shanghai, China) following the manufacturer's instructions. Glycated hemoglobin A1c (HbA1c) levels were measured by high-performance liquid chromatography using automatic analyzers ADAMS A1c HA-8180 (Arkray Factory, Kyoto, Japan). Blood urea nitrogen (BUN), uric acid (UA), serum calcium (Ca), triglycerides (TG), serum iron (SI), serum albumin (Alb), and unsaturated iron-binding capacity (UIBC) were measured by colorimetry using Cobas c 701 (Roche, Basel, Switzerland). Serum creatinine was measured by Enzymatic methods using Cobas c 701 (Roche, Basel, Switzerland). Lipid metabolism-related indicators such as total cholesterol (TC), high-density lipoprotein cholesterol (HDL-C), and low-density lipoprotein cholesterol (LDL-C) were measured by enzymatic colorimetry using Cobas c 701 (Roche, Basel, Switzerland). Ferritin was measured by immunoturbidimetry using Cobas c 701 (Roche, Basel, Switzerland). Transferrin saturation (TAST) was computed by dividing serum iron level by total iron-binding capacity which is equal to that of serum iron plus UIBC. N- terminal B- type natriuretic peptide precursor (NT-proBNP) was measured by Electrochemiluminescence using Cobas e 602 (Roche, Basel, Switzerland). Intact parathyroid hormone (iPTH) was measured by Electrochemiluminescence using Cobas e 801 (Roche, Basel, Switzerland). C-reactive protein (CRP) was measured by the immunoturbidimetry method using a Cobas c702 (Roche, Basel, Switzerland).

### Statistical Analysis

Patient characteristics were described by mean ± *SD*, medians, interquartile ranges, and percentages according to data type. If the data are in accordance with normal distribution, an independent sample *t*-test is used for comparison between the two groups. If the data do not conform to the normal distribution, the Mann-Whitney U test is used for comparison between the two groups. Logistic regression analysis was used to estimate the risk factors of RLS. Receiver-operating characteristic (ROC) analysis and the area under the ROC curve (AUC) were used to identify significant predictors of RLS. To assess the correlation between the severity of RLS and related laboratory indicators, Pearson's correlation coefficient for normally distributed data or Spearman's rank correlation coefficient for non-normally distributed data was used. Thereafter multivariate linear regression analysis was used to adjust confounding factors. The variables of skewed distributions including duration of PD, hepcidin, and RKF were log-transformed to obtain normal distributions before multivariate linear regression analyses. All of these data were analyzed using the SPSS 23 software package(IBM SPSS Statistics for Windows 2015, IBM Corp, New York, USA) and *P*-values were calculated as two-sided. Statistical significance was set at *P* < 0.05.

## Results

### Demographic Characteristics

The demographic data and laboratory analysis results of the 153 patients undergoing PD with RLS or without RLS are shown in Table 1. The average age of patients undergoing PD with RLS was 42.76 ± 12.69 years with a male/female ratio of 31:20. The average age of patients undergoing PD without RLS was 46.21 ± 11.04 years with a male/female ratio of 69/33. No age, gender, or body mass index (BMI) differences were noted between the two groups. SBP, DBP, FPG, HbA1C, BUN, Scr, UA, CRP, and lipid index were also similar in the two groups. There were no significant differences in terms of iron metabolism parameters and Kt/V between the two groups. Among the parameters, duration of PD, serum hepcidin level, and calcium level were found to be significantly higher in patients undergoing PD with RLS than those in patients undergoing PD without RLS (*P* < 0.001, *P* < 0.001, and *P* = 0.002, respectively). While, the level of hemoglobin, albumin, and RKF were significantly lower in patients undergoing PD with RLS (*P* = 0.002, *P* = 0.042, and *P* < 0.001, respectively). No significant differences were identified in the other parameters (Table 1).

**Table 1:** Demographic characteristics of patients undergoing PD.

**Patient characteristics**	**RLS (–) (*n* = 102)**	**RLS (+) (*n* = 51)**	***P***
Gender (male/female)	69/33	31/20	0.400
Age (year)	42.76 ± 12.69	46.21 ± 11.04	0.086
Duration of PD (months)	20.33 (5.75, 36.00)	45.00 (22.00, 56.00)	<0.001^*^
BMI (kg/m^2^)	23.69 ± 3.61	23.55 ± 3.63	0.814
SBP (mmHg)	137.82 ± 23.48	141.86 ± 21.658	0.305
DBP (mmHg)	84.20 ± 12.04	86.88 ± 14.30	0.224
Hb (g/L)	101.56 ± 16.97	91.36 ± 21.14	0.002^*^
FPG (mmol/L)	4.31 ± 0.68	4.36 ± 0.62	0.695
HbA1C (%)	5.37 ± 0.53	5.23 ± 0.38	0.189
TCHO (mmol/L)	3.69 ± 1.04	3.86 ± 1.34	0.404
TG (mmol/L)	1.24 ± 0.70	1.35 ± 0.84	0.412
HDL-C(mmol/L)	2.18 ± 1.02	2.20 ± 1.09	0.898
LDL-C(mmol/L)	1.26 ± 0.62	1.48 ± 0.70	0.061
BUN (mmol/L)	29.07 ± 9.80	29.62 ± 11.16	0.758
Scr (μmol/L)	865.53 ± 288.57	901.46 ± 339.54	0.499
UA (μmol/L)	429.46 ± 134.20	449.17 ± 139.85	0.401
K (mmol/L)	4.65 ± 0.77	4.45 ± 0.87	0.156
Ca (mmol/L)	2.06 ± 0.27	2.21 ± 0.25	0.002^*^
P (mmol/L)	1.98 ± 0.53	2.02 ± 0.60	0.697
iPTH (pg/ml)	109.50 (61.34, 419.00)	222.50 (112.52, 389.50)	0.586
Alb (g/L)	35.42 ± 5.05	31.73 ± 4.52	0.042^*^
NT-proBNP (pg/ml)	1056.11 (249.04, 4267.00)	9713.00 (2228.13, 30529.50)	0.182
Kt/V	1.69 ± 0.42	1.73 ± 0.37	0.756
SI (μmol/L)	12.96 ± 6.47	14.33 ± 4.33	0.077
SF (ng/ml)	226.00 (77.95, 380.55)	318.20 (133.65, 478.45)	0.162
TIBC(μmol/L)	41.16 ± 7.44	41.07 ± 9.86	0.956
TSAT (%)	27.81 (21.59, 38.56)	33.23 (26.50, 46.47)	0.189
Hepcidin (ng/ml)	29.40 (20.45, 46.86)	50.40 (30.43, 83.50)	<0.001^*^
CRP (mg/L)	3.00 (1.50, 6.48)	3.49 (2.10, 6.67)	0.176
RKF (ml/min/1.73 m^2^)	3.28 (1.72, 4.75)	0.85 (0.14, 2.70)	<0.001^*^

*Data presented as median (first-third interquartile range) or mean ± SD or number (percentage).*

* *P < 0.05. BMI, body mass index; SBP, systolic blood pressure; DBP, diastolic blood pressure; Hb, hemoglobin; FBG, fasting plasma glucose; HbA1c, glycated hemoglobin A1c; TC, total cholesterol; TG, triglycerides; HDL-C, high-density lipoprotein cholesterol; LDL-C, low-density lipoprotein cholesterol; BUN, blood urea nitrogen; Scr, serum creatinine; UA, uric acid; Ca, serum calcium; P, serum phosphorus; iPTH, intact parathyroid hormone; Alb, serum albumin; NTproBNP, N-terminal B-type natriuretic peptide precursor; SI, serum iron; SF, serum ferritin, TIBC, total iron binding capacity; TAST, transferrin saturation*.

### Risk Factors Associated With RLS in Patients Undergoing PD

The risk factors were analyzed in two groups: patients undergoing PD with or without RLS. The univariate analysis showed that there was a significant difference in duration of PD [odds ratio (OR) 1.033, 95% CI: 1.017, 1.05, *P* < 0.001], hemoglobulin level (OR 0.97, 95% CI: 0.951, 0.989, *P* = 0.002), calcium level (OR 8.176, 95% CI: 2.062, 32.424, *P* = 0.003), albumin level (OR 0.86, 95% CI: 0.798, 0.927, *P* < 0.001), hepcidin level (OR 1.026, 95% CI: 1.013, 1.039, *P* < 0.001), and RKF (OR 0.613, 95% CI: 0.493, 0.763, *P* < 0.001) between the two groups. We next applied multivariable logistic regression analysis including all of the factors that were significantly different on univariate analysis and found that duration of PD (OR 1.038, 95% CI: 1.017, 1.06, *P* < 0.001), hemoglobulin level (OR 0.969, 95% CI: 0.944, 0.995, *P* = 0.019), calcium level (OR 9.224, 95% CI: 1.261, 67.45, *P* = 0.029), albumin level (OR 0.835, 95% CI: 0.757, 0.921, *P* < 0.001), hepcidin level (OR 1.023, 95% CI: 1.009, 1.038, *P* = 0.001), and RKF (OR 0.65, 95% CI: 0.495, 0.856, *P* = 0.002) were independent determinant factors for RLS in patients undergoing PD (Table 2).

**Table 2:** Univariate analysis and multivariate logistic regression model for RLS in PD patients.

**Variable**	**Univariate analysis**		**Multivariate analysis**	
	**OR (95% CI)**	***P***	**OR (95% CI)**	***P***
Duration of PD (months)	1.033 (1.017, 1.050)	<0.001^*^	1.038 (1.017, 1.060)	<0.001^*^
Hb (g/L)	0.970 (0.951, 0.989)	0.002^*^	0.969 (0.944, 0.995)	0.019^*^
Ca (mmol/L)	8.176 (2.062, 32.424)	0.003^*^	9.224 (1.261, 67.450)	0.029^*^
P (mmol/L)	0.889 (0.495, 1.597)	0.695		
iPTH (pg/ml)	1.001 (1.000, 1.002)	0.225		
SI (μmol/L)	1.040 (0.982, 1.101)	0.177		
SF (ng/ml)	1.000 (0.999, 1.001)	0.562		
TIBC (μmol/L)	0.999 (0.959, 1.040)	0.949		
TSAT (%)	1.013 (0.993, 1.033)	0.198		
Alb (g/L)	0.860 (0.798, 0.927)	<0.001^*^	0.835 (0.757, 0.921)	<0.001^*^
Hepcidin (ng/ml)	1.026 (1.013, 1.039)	<0.001^*^	1.023 (1.009, 1.038)	0.001^*^
Kt/V	1.300 (0.566, 2.989)	0.536		
RKF (ml/min/1.73 m^2^)	0.613 (0.493, 0.763)	<0.001^*^	0.650 (0.495, 0.856)	0.002^*^

* *P < 0.05.*

*Hb, hemoglobin; Ca, serum calcium; P, serum phosphorus; iPTH, intact parathyroid hormone; Alb, serum albumin; SI, serum iron; SF, serum ferritin, TIBC, total iron binding capacity; TAST, transferrin saturation; RKF, residual kidney function*.

### Effect of Serum Hepcidin and Other Factors on Prediction of RLS in Patients Undergoing PD

**Figure 1:**
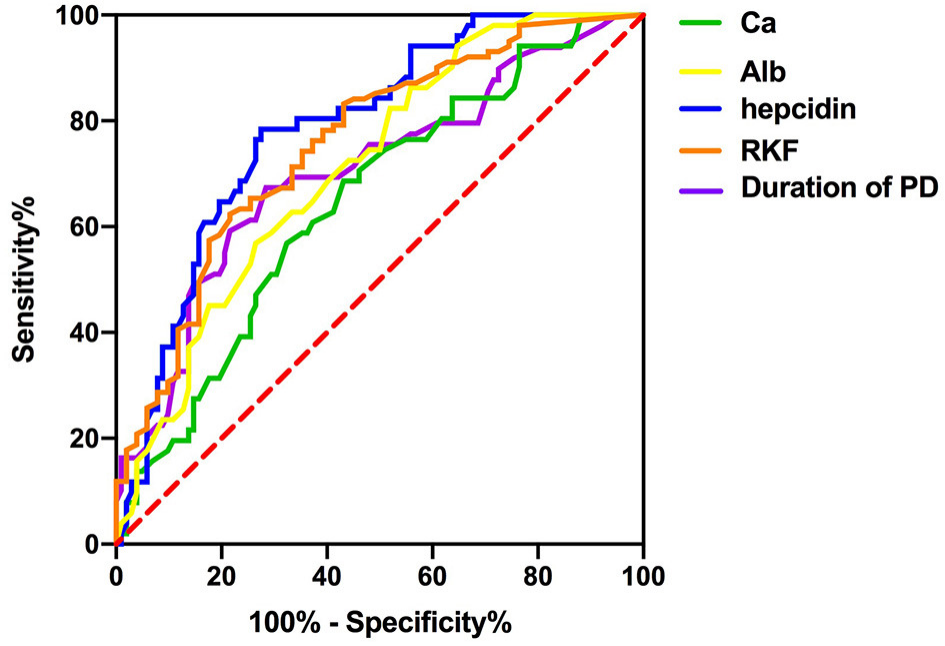
ROC curves for serum hepcidin, Ca, Alb, duration of PD, and RKF.

To investigate the predictive power of serum hepcidin and other significant factors in the diagnosis of RLS in patients undergoing PD, we determined the area under the ROC curve (AUC) by using a ROC analysis. We compared the discrimination of five predictors for RLS in patients undergoing PD in Table 3 and Figure 1, which showed that serum hepcidin has the highest AUC among the predictors. ROC analyses indicated that serum hepcidin yielded an AUC of 0.787 (95% CI: 0.714–0.86, *P* < 0.001). The optimal serum hepcidin cut-off point to predict RLS was 105.78 ng/ml (66.7% sensitivity; 89.2% specificity) (Figure 1). The ROC analyses of calcium, albumin, duration of PD, and RKF were shown in Table 3 and Figure 1.

**Table 3:** ROC analyses of five predictors for RLS in patients undergoing PD.

**Predictor**	**AUC**	**95%CI**	***P***	**Optimal cut-off value**	**Sensitivity%**	**Specificity%**
Serum hepcidin (ng/ml)	0.787	0.714, 0.860	<0.001	105.78	66.7	89.2
Ca (mmol/L)	0.648	0.558, 0.738	0.003	2.11	68.6	56.9
Alb (g/L)	0.712	0.630, 0.794	<0.001	32.85	58.8	71.5
Duration of PD (months)	0.705	0.613, 0.896	<0.001	33.00	67.3	71.6
RKF (ml/min/1.73m^2^)	0.757	0.675, 0.838	<0.001	2.75	62.3	78.4

*Ca, serum calcium; Alb, serum albumin; AUC, area under curve; RKF, residual kidney function*.

### Risk Factors Associated With the Severity of RLS in Patients Undergoing PD

The correlation of all parameters with the IRLS score was evaluated using Spearman's correlation test or Pearson's correlation test, and we found that duration of PD, serum hepcidin level, and calcium level were positively correlated with IRLS score (Spearman's correlation: Rho = 0.347, *P* < 0.001, Spearman's correlation: Rho = 0.494, *P* < 0.001, and Pearson's correlation: *r* = 0.27, *P* = 0.001, respectively). The level of hemoglobulin, albumin, and RKF were negatively correlated with IRLS score (Pearson's correlation: *r* = −0.288, *P* < 0.001; Pearson's correlation: *r* = −0.249, *P* = 0.002; and Spearman's correlation: Rho = −0.478, *P* < 0.001; respectively) (Table 4). A multivariate linear regression analysis was performed on the factors determined to have a relation with the IRLS score in bivariate correlation test. The variables of skewed distributions including duration of PD, hepcidin, and RKF were log transformed to reduce the skewness of the data before multivariate linear regression analyses. The results showed that Log_10_ (duration of PD) (b: 4.275, 95% CI: 1.584, 6.996, *P* = 0.002), Log_10_ hepcidin (b: 12.992, 95% CI: 8.079, 17.906, *P* < 0.001), calcium level (b: 5.153, 95% CI: 0.181, 10.125, *P* = 0.042) were positively associated with IRLS score, whereas hemoglobulin level (b: −0.12, 95% CI: −0.19, −0.049, *P* = 0.001) and Log_10_ RKF (b: −3.795, 95% CI: −7.008, −0.581, *P* = 0.021) were negatively correlated with IRLS score (Table 5).

**Table 4:** Correlation analysis of factors associated with the IRLS score in patients undergoing PD.

**Variable**	**Spearman's rho/Pearson r**	***P***
Duration of PD (months)	0.347	<0.001^*^
Hb (g/L)	−0.288	<0.001^*^
Ca (mmol/L)	0.270	0.001^*^
P (mmol/L)	−0.048	0.555
iPTH (pg/ml)	0.107	0.197
SI (μmol/L)	0.087	0.286
SF (ng/ml)	0.025	0.759
TIBC (μmol/L)	0.063	0.440
TSAT (%)	0.043	0.634
Alb (g/L)	−0.249	0.002^*^
Hepcidin (ng/ml)	0.494	<0.001^*^
Kt/V	0.076	0.353
RKF (ml/min/1.73 m^2^)	−0.478	<0.001^*^

*Spearman's rank correlation was used for the correlation of non- normal distributed data including duration of PD, iPTH, SF, TAST, hepcidin, and RKF. Pearson's correlation coefficient was used for the correlation analysis of normally distributed data including Hb, Ca, P, SI, TIBC, Alb, and Kt/V.*

* *P < 0.05. Hb, hemoglobin; Ca, serum calcium; P, serum phosphorus; iPTH, intact parathyroid hormone; Alb, serum albumin; SI, serum iron; SF, serum ferritin, TIBC, total iron binding capacity; TAST, transferrin saturation; RKF, residual kidney function*.

**Table 5:** Multivariate linear regression analysis of factors associated with the IRLS score in patients undergoing PD.

**Variable**	**b**	**95%CI**	***t***	***P***
Log_10_ (duration of PD)	4.275	1.584, 6.966	3.143	0.002^*^
Hb (g/dL)	−0.120	−0.190, −0.049	−3.350	0.001^*^
Ca (mg/dL)	5.153	0.181, 10.125	2.050	0.042^*^
Alb (g/L)	−0.213	−0.473, 0.047	−1.623	0.107
Log_10_ Hepcidin	12.992	8.079, 17.906	5.231	<0.001^*^
Log_10_ RKF	−3.795	−7.008, −0.581	−2.336	0.021

*The variables of skewed distributions including duration of PD, hepcidin, and RKF were log transformed to reduce the skewness of the data before multivariate linear regression analyses.*

* *P < 0.05. Hb, hemoglobin; Ca, serum calcium; Alb, serum albumin; RKF, residual kidney function*.

## Discussion

As a sleep-related sensorimotor disorder, RLS can be divided into primary RLS and secondary RLS (19). RLS caused by ESRD is one of the most common secondary RLS (20). The clinical manifestation is unbearable discomfort in the deep part of both lower limbs, such as ant walking, needling, itching, and so on (20, 21). It is generally bilateral symmetry, some of which can involve the thigh or upper limb, which is more common at night. Forced movement of the limbs is often needed to achieve relief. RLS seriously affects the life quality and prognosis of patients. Several studies had focused on pharmacological and nonpharmacological treatments of RLS and showed that gabapentin is the most effective pharmacologic intervention which also might improve sleep quality (22). However, there is still a lack of effective treatment measure (23), and it's important to understand the pathogenesis of RLS.

At present, it is believed that iron deficiency and dopamine dysfunction are the main pathogenesis of RLS. Nordlander first proposed that abnormal iron metabolism may be an important pathogenesis of RLS (24). After that, clinical studies, biochemical experiments, pathology, and imaging studies have found that RLS is related to abnormal iron metabolism and abnormal function of the dopaminergic system (25). Iron is a cofactor of tyrosine hydroxylase, which regulates tyrosine metabolism and affects dopamine synthesis (26). The decrease of iron in the brain of patients with RLS can down-regulate the function of the dopamine energy system, resulting in an increase in excitability of the spinal cord, resulting in symptoms of RLS (27). However, due to the increase of serum ferritin secondary to inflammation and intravenous or oral iron supplements in ESRD patients, the level of serum ferritin does not fully reflect decreased iron stores in these patients (7).

Hepcidin is the main substance that regulates iron metabolism and acts on membrane iron transporter-1 on the reticuloendothelial system, duodenal epithelial cells, hepatocytes, and other cell membranes (28). Hepcidin can internalize and degrade iron, reduce the absorption of oral iron, and release stored iron. At last, hepcidin will induce an increase in intracellular iron storage and a decrease in circulating iron levels, which in turn leads to a decrease in the availability of iron in circulation (28). Some studies have shown that hepcidin and its receptors also exist in brain tissue and it can also participate in the regulation of iron metabolism in the brain (29).

Studies on primary RLS showed that the level of serum hepcidin in RLS patients was higher than that in healthy controls, but there was no difference in serum ferritin levels. High serum hepcidin levels were associated with the age of onset of RLS, RLS positive family history, and decreased sleep. It is speculated that compared with serum ferritin serum hepcidin is a biomarker that can better reflect RLS (13). Another study on primary RLS shows that serum hepcidin level is positively correlated with periodic leg movement, while serum ferritin is not (30). It is considered that RLS patients have iron homeostasis disorder, not an absolute iron deficiency, which may be similar to functional deficiency (12, 13). High levels of serum ferritin limit the availability of iron and its redistribution to different organs, including the brain. Ahmet Tufekci et al. found that serum hepcidin levels were positively correlated with the severity of RLS on hemodialysis patients (14). While, so far, there is no study on the relationship between hepcidin and RLS in patients undergoing PD. Our study evaluates the relation between RLS and hepcidin in patients undergoing PD for the first time. The results demonstrated an independent relation between RLS and hepcidin in patients undergoing PD and indicate that a serum hepcidin level above 105.78 ng/ml may have a positive predictive value for the presence of RLS.

Previous studies have found that the duration of hemodialysis is a risk factor for RLS, and the RLS severity was positively correlated with the duration of dialysis (31, 32). Our studies have produced consistent results. Our results showed that the duration of dialysis in the RLS group was 42.63 ± 26.82 months, which was significantly longer than that in the non-RLS group. However, previous studies have not suggested that the duration of PD is an independent risk factor for RLS in patients undergoing PD (33, 34), so more studies are needed to further confirm it.

Our study found that hypercalcemia is a risk factor for RLS in patients with PD. Similar to the result, previous studies showed that the calcium of RLS patients was significantly higher than the control group and positively related to the severity of RLS (35, 36). It is not clear how hypercalcemia leads to the occurrence and development of RLS. We speculate that RLS may be associated with vascular calcification and vascular dysfunction caused by hypercalcemia (37). Hypercalcemia is a manifestation of chronic kidney disease-mineral and bone disorder (CKD-MBD) in patients undergoing PD. In addition to hypercalcemia, phosphorus metabolism disorders and secondary hyperparathyroidism often exist in patients undergoing PD, which had been demonstrated to be related to the pathogenesis of RLS in patients with ESRD (38, 39). However, in our study, we did not find that the levels of serum phosphorus and parathyroid hormone were associated with RLS. In the future, more studies are needed to further confirm it. In general, prevention and aggressive treatment of CKD-MBD may help prevent or reduce symptoms associated with RLS.

Hypoalbuminemia and anemia in hemodialysis patients have been reported to be risk factors for RLS (31, 40), and our results are consistent with this in this study. Anemia is not only an indicator of malnutrition but may also be the result of functional iron deficiency which also induced RLS. In ESRD patients undergoing hemodialysis with RLS, the occurrence of RLS is closely related to reduced /absent RKF (41), which is consistent with our study. The deterioration of RKF may aggravate anemia, inflammation, and malnutrition (42). Besides that, RKF could also affect the clearance of small and medium molecules (43), which may be a vital mechanism of RLS.

To sum up, RLS in patients undergoing PD is affected by many factors, among which hepcidin is especially important. A high level of serum ferritin is not only an independent risk factor of RLS and positively correlated with the severity of RLS. Hepcidin is expected to be a new therapeutic target for RLS in patients undergoing PD.

## Conclusion

The results of the present study show that a significant relationship exists between increased serum hepcidin level and RLS in patients undergoing PD. A serum hepcidin level above 105.78 ng/ml may be a positive predictive factor for the presence of RLS. We suggest that the evaluation of serum hepcidin levels in patients undergoing PD with complex iron metabolisms may contribute to a diagnosis of accompanying RLS. Future studies are required on this subject.

## Data Availability Statement

The raw data supporting the conclusions of this article will be made available by the authors, without undue reservation.

## Ethics Statement

Written informed consent was obtained from the individuals for the publication of any potentially identifiable images or data included in this article.

## Author Contributions

YG, LT, and PL: study design and manuscript preparation. YS, TP, XL, YW, LY, YL, and LW: literature search, data collection, statistical analysis, and data interpretation. All authors contributed to the article and approved the submitted version.

## Funding

This work was supported by the Joint Construction Project of Henan Province (No. LHGJ20190245).

## Conflict of Interest

The authors declare that the research was conducted in the absence of any commercial or financial relationships that could be construed as a potential conflict of interest.

## Publisher's Note

All claims expressed in this article are solely those of the authors and do not necessarily represent those of their affiliated organizations, or those of the publisher, the editors and the reviewers. Any product that may be evaluated in this article, or claim that may be made by its manufacturer, is not guaranteed or endorsed by the publisher.

## References

[1] Li P Chow K Van de Luijtgaarden M Johnson D Jager K Mehrotra R. Changes in the worldwide epidemiology of peritoneal dialysis. Nat Rev Nephrol. (2017) 13:90–103. 10.1038/nrneph.2016.181 28029154

[2] de Menezes A Motta D de Carvalho F Santana-Santos E de Andrade Júnior M Figueirôa M. Restless legs syndrome in dialysis patients: does the dialysis modality influence its occurrence and severity? Int J Nephrol. (2018) 2018:1414568. 10.1155/2018/1414568 29682346 PMC5845496

[3] Högl B Stefani A. Restless legs syndrome and periodic leg movements in patients with movement disorders: specific considerations. Mov Disord. (2017) 32:669–81. 10.1002/mds.26929 28186669

[4] Trenkwalder C Allen R Högl B Clemens S Patton S Schormair B. Comorbidities, treatment, and pathophysiology in restless legs syndrome. The Lancet Neurology. (2018) 17:994–1005. 10.1016/S1474-4422(18)30311-9 30244828

[5] Ferré S García-Borreguero D Allen R Earley C. New insights into the neurobiology of restless legs syndrome. Neuroscientist. (2019) 25:113–25. 10.1177/1073858418791763 30047288 PMC9372713

[6] Winkelman J. Considering the causes of RLS. Eur. J. Neurol. (2006) 13:8–14. 10.1111/j.1468-1331.2006.01588.x-i1 16930377

[7] Ueda N Takasawa K. Impact of inflammation on ferritin, hepcidin and the management of iron deficiency anemia in chronic kidney disease. Nutrients. (2018) 10:1173. 10.3390/nu10091173 30150549 PMC6163440

[8] Mleczko-Sanecka K Silvestri L. Cell-type-specific insights into iron regulatory processes. Am J Hematol. (2021) 96:110–27. 10.1002/ajh.26001 32945012

[9] Preza G Pinon R Ganz T Nemeth E. Cellular catabolism of the iron-regulatory peptide hormone hepcidin. PLoS ONE. (2013) 8:e58934. 10.1371/journal.pone.0058934 23536837 PMC3594189

[10] Raha A Vaishnav R Friedland R Bomford A Raha-Chowdhury R. The systemic iron-regulatory proteins hepcidin and ferroportin are reduced in the brain in Alzheimer's disease. Acta Neuropathol Commun. (2013) 1:55. 10.1186/2051-5960-1-55 24252754 PMC3893417

[11] Gong J Du F Qian Z Luo Q Sheng Y Yung W. Pre-treatment of rats with ad-hepcidin prevents iron-induced oxidative stress in the brain. Free Radic Biol Med. (2016) 90:126–32. 10.1016/j.freeradbiomed.2015.11.016 26582371

[12] Dauvilliers Y Chenini S Vialaret J Delaby C Guiraud L Gabelle A. Association between serum hepcidin level and restless legs syndrome. Mov Disord. (2018) 33:618–27. 10.1002/mds.27287 29418021

[13] Chenini S Delaby C Rassu A Barateau L Vialaret J Hirtz C. Hepcidin and ferritin levels in restless legs syndrome: a case-control study. Sci Rep. (2020) 10:11914. 10.1038/s41598-020-68851-0 32681031 PMC7367854

[14] Tufekci A Kara E. Relation of serum hepcidin levels and restless legs syndrome in chronic hemodialysis patients. Sleep Breath. (2020). 10.1007/s11325-020-02209-8 33029690

[15] Inker L Astor B Fox C Isakova T Lash J Peralta C. KDOQI US commentary on the 2012 KDIGO clinical practice guideline for the evaluation and management of CKD. Am J Kidney Dis. (2014) 63:713–35. 10.1053/j.ajkd.2014.01.416 24647050

[16] Allen R Picchietti D Hening W Trenkwalder C Walters A Montplaisi J. Restless legs syndrome: diagnostic criteria, special considerations, and epidemiology. A report from the restless legs syndrome diagnosis and epidemiology workshop at the National Institutes of Health. Sleep Med. (2003) 4:101–19. 10.1016/S1389-9457(03)00010-8 14592341

[17] Golper T. A summary of the 2000 update of the NKF-K/DOQI clinical practice guidelines on peritoneal dialysis adequacy. Perit Dial Int. (2001) 21:438–40. 10.1177/089686080102100503 11757825

[18] Walters A LeBrocq C Dhar A Hening W Rosen R Allen R. Validation of the International Restless Legs Syndrome Study Group rating scale for restless legs syndrome. Sleep Med. (2003) 4:121–32. 10.1016/S1389-9457(02)00258-7 14592342

[19] Didato G Di Giacomo R Rosa G Dominese A de Curtis M Lanteri P. Restless legs syndrome across the lifespan: symptoms, pathophysiology, management and daily life impact of the different patterns of disease presentation. Int J Environl Res Public Health. (2020) 17:3658. 10.3390/ijerph17103658 32456058 PMC7277795

[20] Huang C Lee M Wang L Lee P Tu Y Hsu C. Comparative efficacy and acceptability of treatments for restless legs syndrome in end-stage renal disease: a systematic review and network meta-analysis. Nephrol Dial Transplant. (2020) 35:1609–18. 10.1093/ndt/gfz097 31157898

[21] Novak M Winkelman J Unruh M. Restless legs syndrome in patients with chronic kidney disease. Semin Nephrol. (2015) 35:347–58. 10.1016/j.semnephrol.2015.06.006 26355253

[22] Chen J Lee T Tu Y Kuo G Yang H Yen C. Pharmacological and nonpharmacological treatments for restless legs syndrome in end stage kidney disease: a systematic review and component network meta-analysis. Nephrol Dial Transplant. (2021). 10.1093/ndt/gfab290 34612498 PMC9494057

[23] Gonzalez-Latapi P Malkani R. Update on restless legs syndrome: from mechanisms to treatment. Curr Neurol Neurosci Rep. (2019) 19:54. 10.1007/s11910-019-0965-4 31250128

[24] Nordlander N. Therapy in restless legs. Acta Med Scand. (1953) 145:453–57. 10.1111/j.0954-6820.1953.tb07042.x 13079659

[25] Connor J Ponnuru P Wang X Patton S Allen R Earley C. Profile of altered brain iron acquisition in restless legs syndrome. Brain. (2011) 134:959–68. 10.1093/brain/awr012 21398376 PMC3069701

[26] Kaushik P Gorin F Vali S. Dynamics of tyrosine hydroxylase mediated regulation of dopamine synthesis. J Comput Neurosci. (2007) 22:147–60. 10.1007/s10827-006-0004-8 17053993

[27] Connor J Wang X Allen R Beard J Wiesinger J Felt B. Altered dopaminergic profile in the putamen and substantia nigra in restless leg syndrome. Brain. (2009) 132:2403–12. 10.1093/brain/awp125 19467991 PMC2732265

[28] Rauf A Shariati M Khalil A Bawazeer S Heydari M Plygun S. Hepcidin, an overview of biochemical and clinical properties. Steroids. (2020) 160:108661. 10.1016/j.steroids.2020.108661 32450084

[29] Du F Qian Z Luo Q Yung W Ke Y. Hepcidin suppresses brain iron accumulation by downregulating iron transport proteins in iron-overloaded rats. Mol Neurobiol. (2015) 52:101–14. 10.1007/s12035-014-8847-x 25115800

[30] Im H Kim J Yun C Kim D Oh J. Changes in hepcidin serum levels correlate with clinical improvement in idiopathic restless legs syndrome patients. J Clin Med. (2020) 9:4115. 10.3390/jcm9124115 33419264 PMC7766726

[31] Lin C Wu V Li W Sy H Wu S Chang C. Restless legs syndrome in end-stage renal disease: a multicenter study in Taiwan. Eur J Neurol. (2013) 20:1025–31. 10.1111/ene.12095 23369046

[32] Zhang L Ma X Lin J Liu W Guo W Yin L. Prevalence and risk factors of restless legs syndrome in hemodialysis patients. Nat Sci Sleep. (2020) 12:19–27. 10.2147/NSS.S236393 32021521 PMC6970009

[33] Merlino G Lorenzut S Romano G Sommaro M Fontana A Montanaro D. Restless legs syndrome in dialysis patients: a comparison between hemodialysis and continuous ambulatory peritoneal dialysis. Neurol Sci. (2012) 33:1311–8. 10.1007/s10072-012-0953-9 22271263

[34] Losso R Minhoto G Riella M. Sleep disorders in patients with end-stage renal disease undergoing dialysis: comparison between hemodialysis, continuous ambulatory peritoneal dialysis and automated peritoneal dialysis. Int Urol Nephrol. (2015) 47:369–75. 10.1007/s11255-014-0860-5 25358390

[35] Turk A Ozkurt S Turgal E Sahin F. The association between the prevalence of restless leg syndrome, fatigue, and sleep quality in patients undergoing hemodialysis. Saudi Med J. (2018) 39:792–8. 10.15537/smj.2018.8.22398 30106417 PMC6194982

[36] Zadeh Saraji N Hami M Boostani R Mojahedi M. Restless leg syndrome in chronic hemodialysis patients in Mashhad hemodialysis centers. J Renal Injury Prevent. (2017) 6:137–41. 10.15171/jrip.2017.27 28497091 PMC5423282

[37] Levin N Hoenich N. Consequences of hyperphosphatemia and elevated levels of the calcium-phosphorus product in dialysis patients. Curr Opin Nephrol Hypertens. (2001) 10:563–8. 10.1097/00041552-200109000-00003 11496047

[38] Takaki J Nishi T Nangaku M Shimoyama H Inada T Matsuyama N. Clinical and psychological aspects of restless legs syndrome in uremic patients on hemodialysis. Am J Kidney Dis. (2003) 41:833–9. 10.1016/S0272-6386(03)00031-3 12666070

[39] Santos R Coelho F da Silva B Graciolli F Dominguez W de Menezes Montenegro F. Parathyroidectomy Improves Restless Leg Syndrome in Patients on Hemodialysis. PLoS ONE. (2016) 11:e0155835. 10.1371/journal.pone.0155835 27196740 PMC4873141

[40] La Manna G Pizza F Persici E Baraldi O Comai G Cappuccilli M. Restless legs syndrome enhances cardiovascular risk and mortality in patients with end-stage kidney disease undergoing long-term haemodialysis treatment. Nephrol Dial Transplant. (2011) 26:1976–83. 10.1093/ndt/gfq681 21056943

[41] Pizza F Persici E La Manna G Campieri C Plazzi G Carretta E. Family recurrence and oligo-anuria predict uremic restless legs syndrome. Acta Neurol Scand. (2012) 125:403–9. 10.1111/j.1600-0404.2011.01581.x 21824115

[42] Arai Y Shioji S Tanaka H Katagiri D Hinoshita F. A novel uremic score reflecting accumulation of specific uremic toxins more precisely predicts one-year mortality after hemodialysis commencement: a retrospective cohort study. Toxins. (2020) 12:634. 10.3390/toxins12100634 33019590 PMC7601006

[43] Cupisti A Bolasco P D'Alessandro C Giannese D Sabatino A Fiaccadori E. Protection of residual renal function and nutritional treatment: first step strategy for reduction of uremic toxins in end-stage kidney disease patients. Toxins. (2021) 13:289 10.3390/toxins13040289 33921862 PMC8073165

